# Clinical application of TBSRTC in thyroid FNAC: a single-center retrospective study

**DOI:** 10.1186/s12902-026-02493-7

**Published:** 2026-08-17

**Authors:** Yuanyuan Lei, Yanlin Wen, Aiwen Chen, Ya Gao, Jinhui Liu, Dongcun Wang

**Affiliations:** Department of Pathology, National Cancer Center/National Clinical Research Center for Cancer/Cancer Hospital & Shenzhen Hospital, Chinese Academy of Medical Sciences and Peking Union Medical College, 113 Baohe Avenue, Longgang, Shenzhen, Guangdong 518116 China

**Keywords:** Thyroid, Fine-needle aspiration cytology, Bethesda System, Risk of malignancy, False-negative diagnosis, False-positive diagnosis

## Abstract

**Background:**

Thyroid fine-needle aspiration cytology (FNAC) classified according to the Bethesda System for Reporting Thyroid Cytopathology (TBSRTC) is widely used for the preoperative evaluation of thyroid nodules. However, category distribution, observed risk of malignancy (ROM), and diagnostic performance may vary according to referral patterns, disease prevalence, and selective surgical follow-up.

**Methods:**

This single-center retrospective study included 2,316 ultrasound-guided thyroid fine-needle aspiration (FNA) specimens from 1,791 patients evaluated at a tertiary cancer center between 2019 and 2022. Bethesda category distribution was assessed in the entire cohort. Observed ROM was calculated in 1,137 FNA sampling events with matched postoperative histopathology. Binary diagnostic performance was evaluated in 1,068 sampling events after exclusion of 42 category III and 27 category IV sampling events.

**Results:**

Bethesda categories I–VI comprised 49 (2.1%), 762 (32.9%), 150 (6.5%), 89 (3.8%), 191 (8.2%), and 1,075 (46.4%) specimens, respectively. Among the 1,137 nodules with histopathologic follow-up, 156 were benign, 22 were borderline or low-risk neoplasms, and 959 were malignant. When borderline or low-risk neoplasms were classified as nonmalignant, the observed ROM values for categories I–VI were 50.0%, 20.5%, 61.9%, 37.0%, 84.2%, and 99.5%, respectively. Sensitivity, specificity, accuracy, positive predictive value, and negative predictive value were 95.6%, 90.0%, 94.9%, 98.6%, and 74.1%, respectively. False-negative diagnoses were predominantly associated with sampling limitations, including minute malignant foci, stromal fibrosis, thyroid follicular nodular disease, and cystic degeneration. False-positive diagnoses were mainly related to overlapping cytomorphologic features in reactive, inflammatory, hyperplastic, cystic, and treatment-related conditions.

**Conclusions:**

Thyroid FNAC showed high diagnostic performance in this surgically selected tertiary cancer-center cohort. The observed ROM and predictive values were influenced by referral bias, surgical selection, and partial verification and should not be generalized to unselected thyroid FNA populations. Accurate targeting, representative sampling, cautious interpretation of focal atypia, and strict nodule-level cytology–histology correlation may reduce diagnostic discordance.

## Introduction

Thyroid nodules are commonly detected during clinical examination and ultrasonography, whereas only a minority are malignant [1, 2]. Because most nodules do not require surgical treatment, accurate preoperative risk assessment is essential for identifying patients who require intervention while avoiding unnecessary surgery. Fine-needle aspiration cytology (FNAC) is a minimally invasive diagnostic procedure and a central component of the risk-adapted evaluation of thyroid nodules [3, 4]. The Bethesda System for Reporting Thyroid Cytopathology (TBSRTC) provides standardized diagnostic terminology, category-specific estimates of the risk of malignancy (ROM), and clinical management recommendations [5, 6]. Nevertheless, the distribution of Bethesda categories and the observed ROM may differ substantially among institutions because of referral patterns, disease prevalence, sampling technique, interpretive thresholds, and selective availability of postoperative histopathology.

These factors are particularly relevant in tertiary cancer centers, where patients with clinically or radiologically suspicious nodules and known or suspected malignancy are overrepresented. In addition, the recognition of borderline or low-risk thyroid neoplasms in the 2022 World Health Organization (WHO) classification [7] and the updated ROM estimates in the 2023 TBSRTC [6] affect comparisons with earlier studies. The practical causes of false-negative and false-positive diagnoses also remain important targets for institutional quality improvement. The present study therefore aimed to describe the Bethesda category distribution and observed ROM in a large tertiary cancer-center cohort, assess the diagnostic performance of thyroid FNAC in the histologically verified subset, analyze the principal causes of cytology–histology discordance, and identify potential strategies for improving sampling, cytologic interpretation, and nodule-level correlation. The purpose was to provide institution-specific real-world evidence rather than to establish the general validity of an already well-established reporting system.

## Materials and methods

### Study design and population

This retrospective single-center study included consecutive ultrasound-guided thyroid FNA specimens processed in the Department of Pathology, Cancer Hospital & Shenzhen Hospital, Chinese Academy of Medical Sciences and Peking Union Medical College, from January 1, 2019, through December 31, 2022. The descriptive cohort comprised 2,316 FNA specimens from 1,791 patients. No routine FNA specimen obtained during the study period was excluded from the analysis of Bethesda category distribution.

Analysis-specific eligibility criteria were applied. The ROM analysis included aspirated nodules with available postoperative histopathologic confirmation. The binary diagnostic-performance analysis additionally required a definitive cytologic classification; Bethesda categories III and IV were considered indeterminate and were excluded. Surgery was performed according to routine clinical decision-making and was not determined by the study protocol.

Each FNA procedure performed at a different time point was recorded as a separate cytologic sampling event because it generated a separate specimen and diagnostic report. Repeated FNAs of the same nodule could therefore contribute more than one record to the descriptive cohort. This approach may introduce within-patient and within-nodule correlation and is addressed as a study limitation.

### Specimen acquisition and preparation

All thyroid FNA specimens were obtained by experienced ultrasonographers under ultrasound guidance. Aspirated material was placed in ThinPrep CytoLyt solution (Hologic, USA). Liquid-based cytology slides were prepared using the ThinPrep 5000 system, fixed in 95% ethanol for 15 min, and stained using the Papanicolaou method. The remaining samples were prepared into cell blocks using plasma–thrombin method, fixed with 10% neutral-buffered formalin, processed routinely, embedded in paraffin, and stained with hematoxylin and eosin.

### Cytologic classification

Each original FNA specimen was interpreted during routine clinical practice by one of two experienced cytopathologists. One cytopathologist had more than 30 years of experience and the other had more than 10 years of experience. The two cytopathologists interpreted mutually exclusive case sets; therefore, no formal interobserver agreement or between-pathologist performance analysis was performed.

The original cytologic diagnoses were rendered using the six-tier Bethesda framework applicable at the time and were mapped to the corresponding categories of the 2023 TBSRTC for the present analysis: category I, nondiagnostic; category II, benign; category III, atypia of undetermined significance (AUS); category IV, follicular neoplasm; category V, suspicious for malignancy; and category VI, malignant [6]. Because the original diagnoses were made between 2019 and 2022, the 2023 subclassification of AUS into nuclear atypia and other atypia was not applied retrospectively.

### Nodule-level cytology–histology correlation

Each FNA sampling event was treated as an analytical record and was linked to a specific target nodule. In patients with multiple nodules, each target nodule was recorded separately, and each cytologic result was matched one-to-one with the corresponding histopathologic lesion. Repeated FNAs of the same nodule performed at different time points were retained as separate sampling events.

### Histopathologic classification and risk of malignancy

Postoperative histopathologic diagnosis was used as the reference standard. Histopathologic terminology was harmonized with the 2022 WHO classification when supported by the original pathology reports [7]. Non-invasive follicular thyroid neoplasm with papillary-like nuclear features (NIFTP), follicular tumor of uncertain malignant potential (FT-UMP), and well-differentiated tumor of uncertain malignant potential (WDT-UMP) were grouped operationally as borderline or low-risk neoplasms. No hyalinizing trabecular tumor was identified in this cohort.

Two observed ROM estimates were calculated. ROM1 classified borderline or low-risk neoplasms as positive outcomes: ROM1 = (borderline or low-risk neoplasms + malignant tumors) / all nodules with histopathologic follow-up in the corresponding Bethesda category x 100%. ROM2 classified borderline or low-risk neoplasms as nonmalignant outcomes: ROM2 = malignant tumors / all nodules with histopathologic follow-up in the corresponding Bethesda category x 100%. This analytical grouping was not intended to imply biological equivalence between low-risk neoplasms and carcinoma.

The original histopathologic diagnoses were made between 2019 and 2022 using the WHO terminology routinely applied at the institution. Because the 2022 WHO classification had not been consistently implemented throughout the study period, invasive encapsulated follicular variant papillary thyroid carcinoma was not recorded as a separate category and may have been included within the broader group of follicular variant papillary thyroid carcinoma.

### Diagnostic-performance analysis

Of the 1,137 nodules with postoperative histopathologic confirmation, 42 category III nodules and 27 category IV nodules were excluded from the binary diagnostic-performance analysis because these categories were indeterminate. The final binary cohort therefore comprised 1,068 nodules.

Bethesda categories I and II were classified as cytologically negative, and categories V and VI were classified as cytologically positive. Histologically benign lesions were classified as negative, whereas borderline or low-risk neoplasms and malignant tumors were classified as positive. Sensitivity, specificity, accuracy, positive predictive value (PPV), and negative predictive value (NPV) were calculated from the resulting 2 × 2 table.

A false-negative diagnosis was defined as category I or II cytology with a positive matched histopathologic outcome. A false-positive diagnosis was defined as category V or VI cytology with a histologically benign matched lesion.

### Review of discordant cases

The cytologic and histologic slides of all false-negative and false-positive cases were retrospectively reviewed by two senior pathologists. Differences were resolved by consensus. Potential causes were classified as specimen/sampling-related factors or interpretive/diagnostic factors. Concordant cases were not systematically re-reviewed.

### Statistical analysis

Categorical variables are reported as counts and percentages. Continuous demographic variables are summarized as range and median. ROM and diagnostic-performance measures were calculated using the definitions above. No formal interobserver agreement or between-pathologist comparison was performed.

## Results

### Study population and Bethesda category distribution

A total of 2,316 thyroid FNA specimens from 1,791 patients were included. The cohort comprised 480 males and 1,311 females (male-to-female ratio, 1:2.7). Patient age ranged from 9 to 82 years, with a median age of 42 years.

**Fig. 1:**
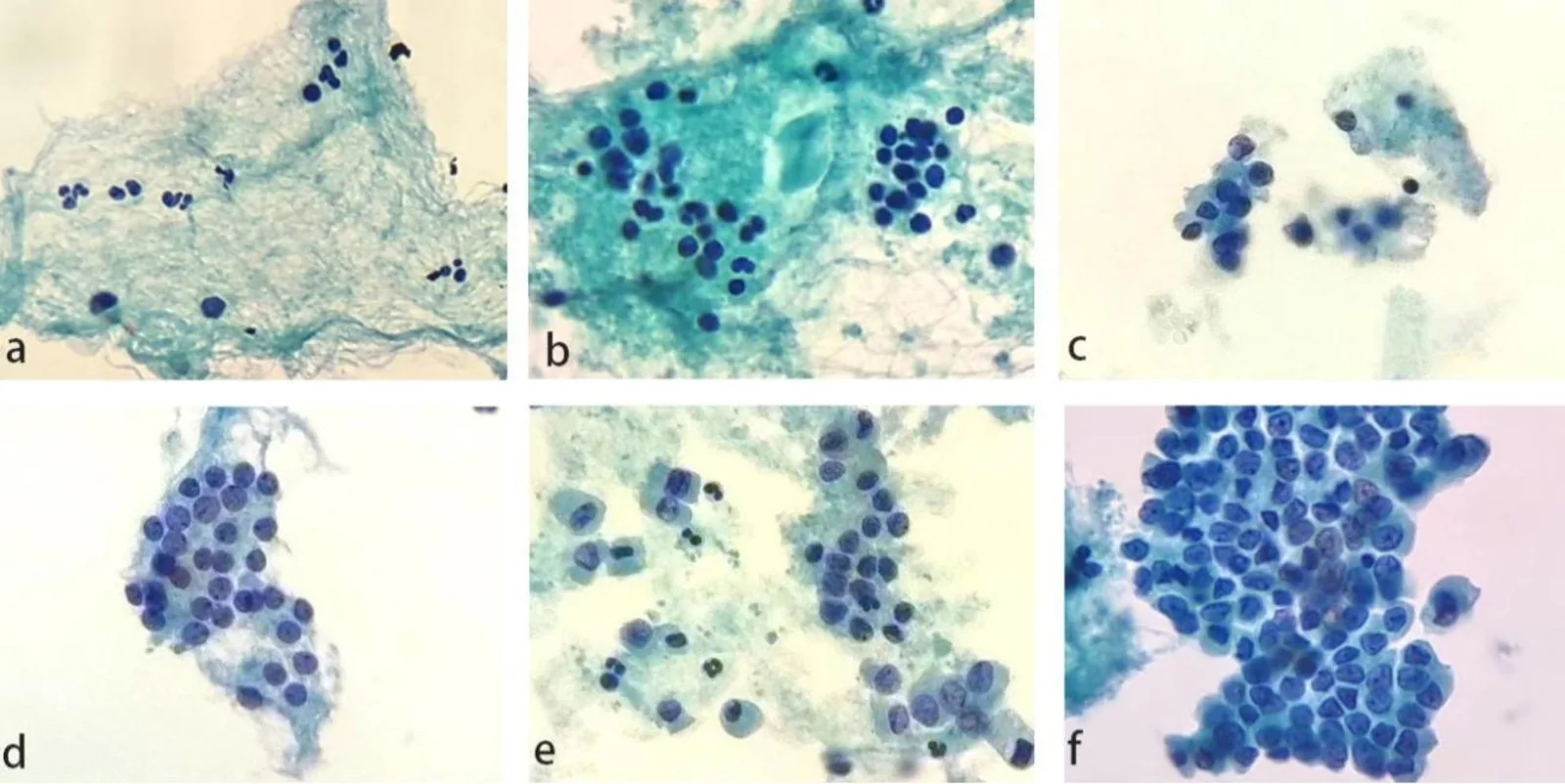
Representative cytomorphologic findings of the six Bethesda categories (Papanicolaou stain, ×400). **a**, Category I: a paucicellular specimen that did not meet the adequacy criteria for follicular epithelial cells. **b**, Category II: monolayered groups of follicular epithelial cells with round, uniformly sized nuclei and finely granular chromatin. **c**, Category III: follicular epithelial cells showing focal nuclear enlargement, chromatin clearing, and mild nuclear membrane irregularity. **d**, Category IV: a cellular specimen composed predominantly of crowded microfollicular groups. **e**, Category V: follicular epithelial cells showing nuclear enlargement, elongation, chromatin clearing, nuclear membrane irregularity, and nuclear grooves. **f**, Category VI: papillary groups of follicular epithelial cells showing nuclear enlargement, chromatin clearing, raisinoid nuclear contour irregularity, nuclear grooves, and intranuclear cytoplasmic pseudoinclusions

Bethesda categories II and VI were the predominant diagnostic categories, accounting for 32.9% and 46.4% of all specimens, respectively. The category distribution was: category I, 49 (2.1%); category II, 762 (32.9%); category III, 150 (6.5%); category IV, 89 (3.8%); category V, 191 (8.2%); and category VI, 1,075 (46.4%). Representative cytomorphologic findings are shown in Fig. 1.

### Histopathologic outcomes and observed risk of malignancy

**Fig. 2:**
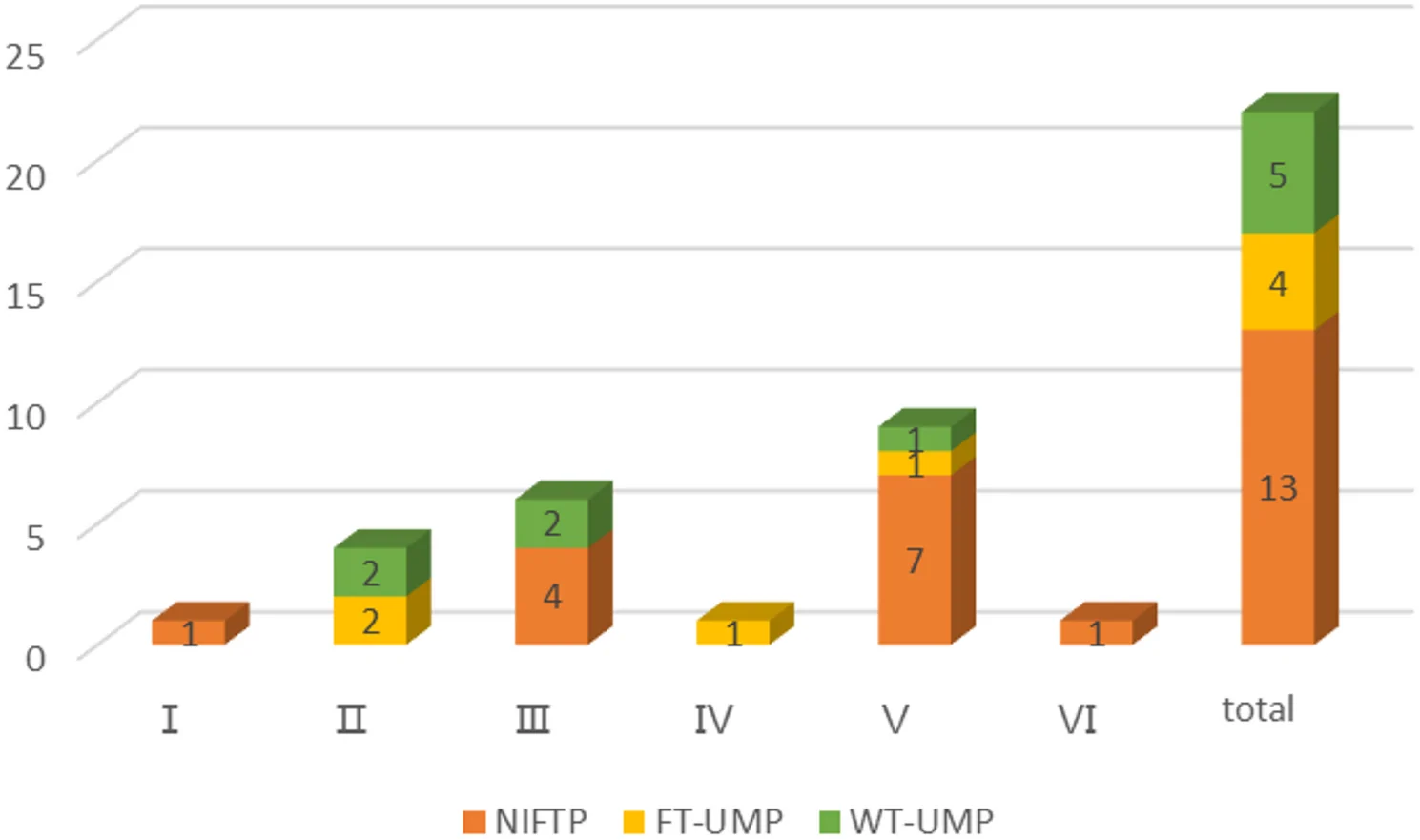
Distribution of borderline or low-risk neoplasms across the Bethesda diagnostic categories. The values above the bars indicate the number of cases in each category

Postoperative histopathologic follow-up was available for 1,137 aspirated nodules (49.1%), including 156 benign lesions, 22 borderline or low-risk neoplasms, and 959 malignant tumors. The 22 borderline or low-risk neoplasms comprised 13 NIFTPs, 4 FT-UMPs, and 5 WDT-UMPs; no HTT was identified. Their distribution across Bethesda categories I–VI was 1, 4, 6, 1, 9, and 1 case, respectively (Fig. 2).

**Fig. 3:**
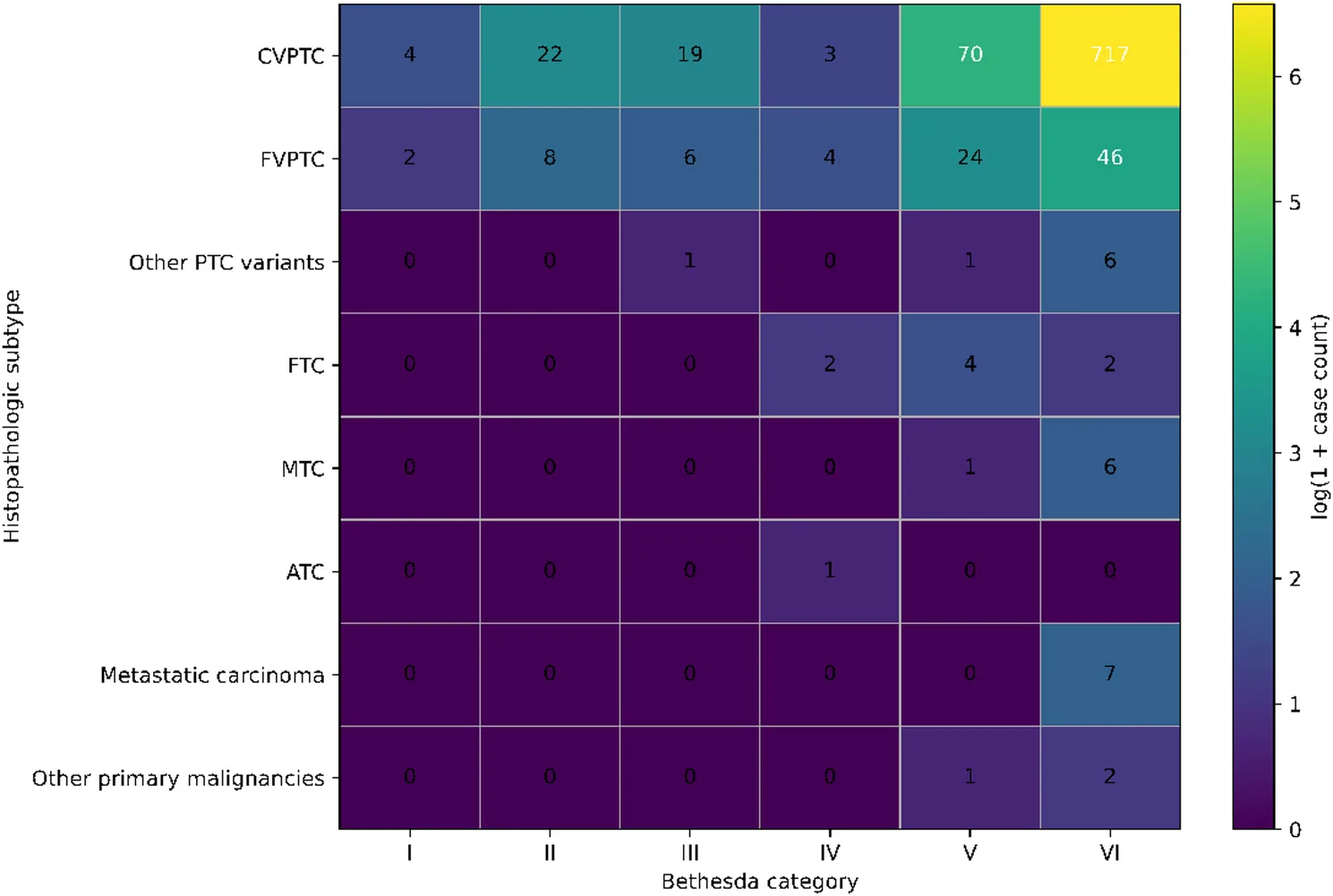
Distribution of malignant histopathologic subtypes across the Bethesda diagnostic categories. A logarithmic color scale was used to improve visualization of less common tumor subtypes; annotated values represent actual case counts. Rows represent histopathologic subtypes, and columns represent Bethesda categories I–VI. The numbers within the cells indicate the actual number of cases. Color intensity represents the relative number of cases, with darker shading indicating a greater number of cases. CVPTC, classic papillary thyroid carcinoma; FVPTC, follicular variant papillary thyroid carcinoma; PTC, papillary thyroid carcinoma; FTC, follicular thyroid carcinoma; MTC, medullary thyroid carcinoma; ATC, anaplastic thyroid carcinoma

Among the 959 malignant tumors, classic papillary thyroid carcinoma was the most common subtype (835 cases), followed by follicular variant papillary thyroid carcinoma (90 cases). The remaining malignancies included 8 other papillary thyroid carcinoma subtypes, 8 follicular thyroid carcinomas, 7 medullary thyroid carcinomas, 1 anaplastic thyroid carcinoma, 7 metastatic carcinomas, and 3 other primary malignant tumors (Fig. 3). Category-specific histopathologic outcomes and ROM values are summarized in Table 1.

**Table 1:** Bethesda category distribution, histopathologic outcomes, and observed risk of malignancy

Bethesda category	FNA sampling events, *n* (%)	Histopathologic follow-up, *n* (%)	Histopathologic outcomes				ROM1		ROM2
			Benign	Borderline/low-risk	Malignancy				
Ⅰ(ND)	49(2.1%)	12(24.5%)	5	1	6		58.3%		50.0%
Ⅱ(Benign)	762(32.9%)	146(19.2%)	112	4	30		23.3%		20.5%
Ⅲ(AUS)	150(6.5%)	42(28.0%)	10	6	26		76.2%		61.9%
Ⅳ(FN)	89(3.8%)	27(30.3%)	16	1	10		40.7%		37.0%
Ⅴ(SFM)	191(8.2%)	120(62.8%)	10	9	101		91.7%		84.2%
Ⅵ(Malignancy)	1075(46.4%)	790(73.5%)	3	1	786		99.6%		99.5%
Total	2316(100%)	1137(49.1%)	156	22	959		86.3%		84.3%

ROM1 classified borderline or low-risk neoplasms as positive outcomes; ROM2 classified them as nonmalignant outcomes

### Diagnostic performance

The binary diagnostic-performance analysis included 1,068 nodules. There were 897 true-positive, 117 true-negative, 13 false-positive, and 41 false-negative results. Sensitivity was 95.6% (897/938), specificity was 90.0% (117/130), accuracy was 94.9% (1,014/1,068), PPV was 98.6% (897/910), and NPV was 74.1% (117/158) (Table 2).

**Table 2:** Cytologic and histopathologic results for 1,068 FNA sampling events with definitive binary cytologic classifications

	Cytology negative	Cytology positive	Total
Histologically negative	117	13	130
Histologically positive	41	897	938
Total	158	910	1068

Bethesda categories III and IV (*n* = 69) were excluded because they were indeterminate. Borderline or low-risk neoplasms were classified as histologically positive in the binary analysis

Bethesda categories III and IV (*n* = 69) were excluded because they were indeterminate. Borderline or low-risk neoplasms were classified as histologically positive in the binary analysis.

### Cytology–histology discordance

The 41 false-negative cases accounted for 3.6% of nodules with histopathologic follow-up (41/1,137), and the 13 false-positive cases accounted for 1.1% (13/1,137). Of the 54 discordant cases, 39 (72.2%) were associated with specimen or sampling-related factors, and 15 (27.8%) were associated with interpretive or diagnostic factors (Table 3).

**Table 3:** Classification of factors associated with 54 false-negative and false-positive diagnoses

	Specimen/sampling-related factors	Interpretive/diagnostic factors	Total
False-negative cases	35	6	41
False-positive cases	4	9	13
Total	39	15	54

**Fig. 4:**
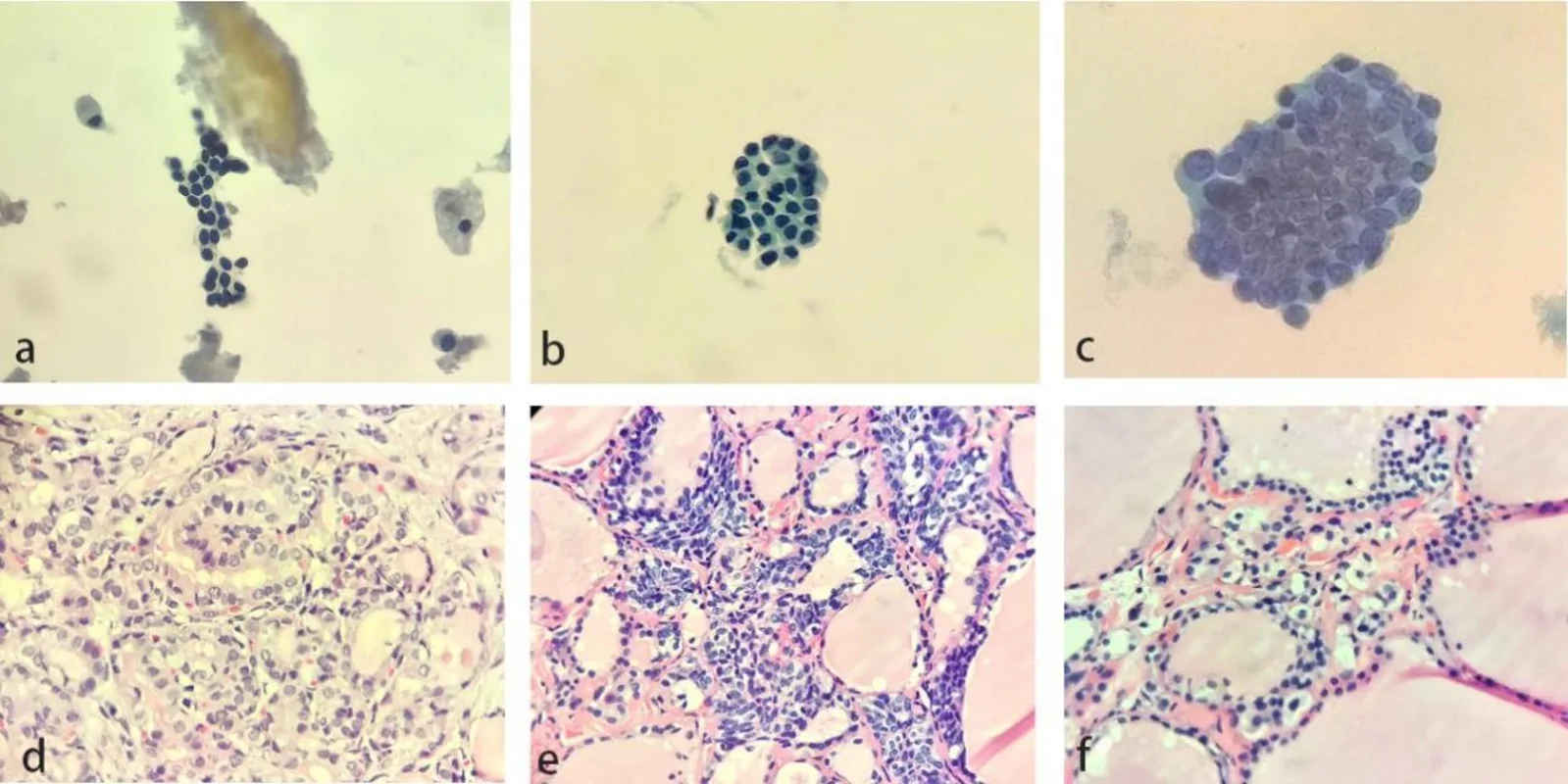
Representative paired cytologic and histopathologic findings from three independent discordant cases. Cytologic images are shown in panels **a**–**c** (Papanicolaou staining, ×400), with the corresponding histologic findings shown in panels **d**–**f** (hematoxylin and eosin staining, ×400). Panels a and d represent Case 1, panels b and e represent Case 2, and panels c and f represent Case 3; the panels do not show sequential disease progression. **a**, Bethesda category II: follicular epithelial cells with round, uniformly sized nuclei, hyperchromatic chromatin, and no definite cytologic atypia. **b**, Bethesda category V: follicular epithelial cells showing nuclear enlargement, chromatin clearing, and irregular nuclear membranes. **c**, Bethesda category VI: a follicular epithelial cell group showing nuclear enlargement, pale chromatin, and focal nuclear contour irregularity. **d**, Histologic examination corresponding to panel a revealed papillary thyroid carcinoma measuring 0.2 cm. **e**, Histologic examination corresponding to panel b revealed a solid cell nest measuring 0.2 cm. **f**, Histologic examination corresponding to panel c showed focal follicular epithelial hyperplasia without a corresponding malignant lesion. Given the small size of the radiologically identified nodule, limitations in gross examination, histologic sampling, or lesion localization could not be excluded

Among the 41 false-negative cases, 35 were attributed primarily to sampling limitations. These included very small malignant foci measuring 3 mm or less (*n* = 28), diffuse stromal fibrosis (*n* = 4), sampling difficulty related to FND (*n* = 2), and cystic degeneration (*n* = 1). Among the 28 cases with malignant foci measuring 3 mm or less, 18 were classic papillary thyroid carcinomas and 10 were follicular variant papillary thyroid carcinomas. In nine cases, a minute carcinoma focus was embedded within a larger target nodule in FND. Although ultrasound-guided FNA sampled the correct target nodule, the aspirated material contained only the surrounding benign follicular nodular component and did not include cells from the minute carcinoma focus. Six false-negative cases were attributed primarily to diagnostic or interpretive factors. Three were low-risk neoplasms whose definitive classification required assessment of capsular or vascular invasion, two reflected diagnostic oversight, and one involved failure to recognize the malignant component in aspirates obtained from bilateral nodules. Nine false-positive cases were attributed primarily to interpretive overlap: follicular epithelial hyperplasia (*n* = 4), thyroiditis (*n* = 3), papillary hyperplasia (*n* = 1), and solid cell nest (*n* = 1). The remaining four cases involved two cystic lesions, one specimen with treatment-related atypia after radiofrequency ablation, and one unresolved 3-mm cytology–histology discrepancy. In the latter case, cytology showed features interpreted as papillary thyroid carcinoma, but no corresponding malignant lesion was identified in the surgical specimen. Failure to identify the very small lesion during gross examination, incomplete histologic representation, or localization limitations could not be excluded. The specific factors are summarized in Table 4, and three representative paired cases are shown in Fig. 4.

**Table 4:** Specific specimen, sampling, and interpretive factors associated with cytology–histology discordance

Factor	False-negative	False-positive	Total
**Specimen/sampling-related factors**			
Very small malignant focus (≤ 3 mm)	28	0	28
Diffuse stromal fibrosis	4	0	4
Sampling difficulty related to FND	2	0	2
Cystic degeneration	1	2	3
Treatment-related atypia after radiofrequency ablation	0	1	1
Unresolved localization or histologic representation of a 3-mm lesion	0	1	1
** Interpretive/diagnostic factors**			
Inability of cytology to assess capsular or vascular invasion	3	0	3
Diagnostic oversight	2	0	2
Failure to recognize a malignant component	1	0	1
Papillary hyperplasia	0	1	1
Follicular epithelial hyperplasia	0	4	4
Solid cell nest	0	1	1
Thyroiditis	0	3	3
**Total**	**41**	**13**	**54**

Among the 28 false-negative cases involving malignant foci measuring 3 mm or less, nine contained a minute carcinoma focus within a larger target nodule in FND; these nine cases are a subset of the 28 and are not counted separately in Table 4.

### Cytomorphologic features of false-positive cases

The four cases of follicular epithelial hyperplasia showed increased cellularity, crowded or overlapping follicular epithelial groups, nuclear enlargement, and variable chromatin clearing. Focal nuclear membrane irregularity and occasional nuclear grooves contributed to overinterpretation. The three thyroiditis cases showed reactive follicular epithelial cells with nuclear enlargement, pale chromatin, and irregular nuclear contours in an inflammatory background. The papillary hyperplasia case contained crowded papillary-like epithelial groups with focal nuclear atypia, whereas the solid cell nest showed cohesive groups of relatively uniform cells with focal nuclear abnormalities. The cystic lesions showed degenerative and reactive epithelial changes, and the post-ablation specimen showed treatment-related cellular atypia. These findings are summarized in Table 5.

**Table 5:** Histopathologic diagnoses and principal cytomorphologic findings in the 13 cases initially classified as false positive

Cause/histopathologic finding	Cases	Principal cytologic findings
Follicular epithelial hyperplasia	4	Increased cellularity, crowded groups, nuclear enlargement, and focal chromatin clearing
Thyroiditis	3	Reactive follicular cells, nuclear enlargement, irregular nuclear contours, and inflammatory background
Papillary hyperplasia	1	Papillary-like groups with focal nuclear atypia
Solid cell nest	1	Cohesive cell groups with focal nuclear abnormalities
Cystic lesions	2	Degenerative and reactive epithelial changes
Post-ablation change	1	Treatment-related nuclear atypia
Unresolved discordance	1	Malignant cytologic interpretation without a corresponding lesion identified histologically

## Discussion

### Overview of the principal findings

The present study evaluated the distribution of Bethesda categories, observed risk of malignancy (ROM), diagnostic performance, and causes of cytology–histology discordance in a large cohort from a tertiary cancer center. The principal findings were the predominance of Bethesda categories II and VI, high sensitivity and positive predictive value in the surgically verified cohort, and a clear difference between the major mechanisms underlying false-negative and false-positive diagnoses. Most false-negative diagnoses were attributable to limitations in lesion representation, particularly minute malignant foci, stromal fibrosis, thyroid follicular nodular disease (FND), and cystic degeneration. In contrast, most false-positive diagnoses resulted from overlap between papillary thyroid carcinoma-like nuclear features and reactive, inflammatory, hyperplastic, cystic, or treatment-related changes. The high observed ROM values in categories I–III and the relatively low negative predictive value should not be interpreted as population-based performance estimates, because the histologically verified cohort was strongly influenced by tertiary referral, surgical selection, and partial verification.

The study does not propose a new classification system and does not establish the general validity of TBSRTC, which is already well supported. Its main contribution is an institution-specific evaluation of how case mix, diagnostic thresholds, selective surgical follow-up, low-risk neoplasm classification, and sampling limitations influence routine thyroid cytology practice. The detailed review of discordant cases also provides practical information that may be useful for local quality improvement and for comparison with other tertiary cancer-center cohorts.

### Distribution of Bethesda categories

Category I accounted for 2.1% of all specimens. This proportion was similar to the 2.5%–3.0% reported in some institutional series [8, 9] but lower than the 8.2%–33.8% described in others [10–12]. Differences in nondiagnostic rates may reflect variation in patient selection, ultrasound guidance, operator experience, adequacy criteria, slide preparation, and the availability of repeat sampling. In our institution, all procedures were performed under ultrasound guidance, liquid-based cytology was used routinely, and residual material was used for cell-block preparation when sufficient material was available. These practices may have contributed to cellular recovery and specimen adequacy, although the retrospective design does not permit a direct causal assessment [11–14].

Categories II and VI were the predominant categories, representing 32.9% and 46.4% of specimens, respectively. The proportion of category VI was substantially higher than that reported in many general-hospital cohorts, but it was comparable with reports from cancer-center settings [8, 15, 16]. This distribution is consistent with the referral profile of our institution, where many patients are evaluated after a suspicious thyroid lesion has already been detected clinically or radiologically. The high proportion of category VI should therefore be interpreted primarily as a feature of case selection rather than as evidence of a general difference in thyroid cytology practice. Correspondingly, the proportion of benign cytology was lower than would be expected in an unselected population undergoing thyroid FNA.

Categories III, IV, and V accounted for 6.5%, 3.8%, and 8.2% of specimens, respectively. The category III proportion was within the range recommended by the 2023 TBSRTC [6]. The relatively low category IV proportion may reflect the small number of aspirates showing a predominantly microfollicular pattern without sufficiently developed papillary thyroid carcinoma-like nuclear features. The category V proportion may also have been affected by local interpretive thresholds: specimens with unequivocal malignant features were generally classified directly as category VI, whereas specimens with only focal or incomplete atypia were more likely to be assigned to category III. These findings illustrate that category distribution is shaped by the interaction between referral patterns, tumor spectrum, preparation method, and institutional thresholds rather than by a single technical factor.

### Observed risk of malignancy

The most striking finding was the high observed ROM in Bethesda categories I–III. When borderline or low-risk neoplasms were classified as nonmalignant, ROM2 was 50.0% for category I, 20.5% for category II, and 61.9% for category III. These values were considerably higher than the reference ranges in the 2023 TBSRTC [6]. However, the denominators were restricted to nodules that underwent surgery. Only 12 of 49 category I nodules and 146 of 762 category II nodules had postoperative histopathologic confirmation. In routine practice, most patients with nondiagnostic or benign cytology are managed with repeat FNA or clinical and ultrasonographic surveillance. Those who proceed to surgery are more likely to have suspicious imaging findings, interval growth, compressive symptoms, discordant clinical findings, or other concerns. The surgically verified category I and II groups were therefore highly selected and enriched for malignancy. The high observed ROM values were likely driven predominantly by surgical selection and partial verification bias. Postoperative histopathologic confirmation was available mainly for nodules that proceeded to surgery because of suspicious ultrasonographic or clinical findings, interval growth, compressive symptoms, or other clinical indications. Consequently, the surgically verified nodules in categories I–III were enriched for malignancy and were not representative of all nodules assigned to these categories. Similar patterns of selective surgical management have been reported in Asian thyroid practice [17–20]. Therefore, the present values should be interpreted as observed ROM estimates among surgically resected nodules rather than population-level ROM estimates.

The category III ROM2 of 61.9% was also high. Surgical selection is particularly important in this category because management is individualized and may include repeat FNA, molecular testing, surveillance, or surgery. In a tertiary cancer center, category III nodules selected for surgery are unlikely to be representative of all AUS nodules. The absence of routine molecular testing during the study period may also have influenced which indeterminate nodules proceeded directly to surgery. The present ROM should consequently be reported as the observed ROM among surgically resected category III nodules rather than as an estimate applicable to the overall category III population.

The category IV ROM2 was 37.0%, which was slightly above the 2023 TBSRTC reference range but broadly compatible with many published surgical series. Only 27 category IV nodules had histopathologic follow-up, and the resulting estimate is therefore imprecise. Follicular-patterned lesions also remain a particular challenge because cytology cannot demonstrate capsular or vascular invasion. Accordingly, the boundary between benign follicular-patterned lesions, tumors of uncertain malignant potential, and follicular carcinoma can be established only after adequate histologic examination of the tumor-capsule interface.

The category V and VI ROM2 values were 84.2% and 99.5%, respectively. The category V value was only marginally above the upper 2023 TBSRTC reference value of 83%, whereas the category VI value was within the recommended range of 97%–100% [6]. The high values are compatible with the cancer-center setting and with the high prevalence of papillary thyroid carcinoma in the surgically verified cohort. Differences in interpretive thresholds may also contribute: cases with fully developed malignant features were classified as category VI, while lesions with incomplete but concerning papillary thyroid carcinoma-like nuclear changes were assigned to category V or, when the changes were more limited, category III. Therefore, comparisons of category V ROM among institutions should consider differences in case mix, preparation method, and the threshold separating AUS, suspicious for malignancy, and malignant categories.

### Diagnostic performance and the low negative predictive value

After excluding categories III and IV, FNAC showed a sensitivity of 95.6%, specificity of 90.0%, accuracy of 94.9%, PPV of 98.6%, and NPV of 74.1%. The high sensitivity, accuracy, and PPV indicate that a category V or VI result in this surgically selected cohort was strongly associated with a positive histopathologic outcome. However, these measures must be interpreted in relation to the analytical definitions and disease prevalence. Borderline or low-risk neoplasms were classified as histologically positive in the binary analysis, and the cohort contained a very high proportion of malignant and low-risk outcomes.

The NPV was lower than that reported in many unselected FNA populations. This finding does not mean that category I or II cytology has an NPV of 74.1% in routine clinical practice. Diagnostic performance was calculated only among nodules with postoperative histopathologic confirmation. Most benign cytology nodules were not resected and were therefore absent from the denominator, whereas negative cytology nodules that underwent surgery were clinically selected because of persistent concern. This partial verification and surgical selection enriched the cytology-negative surgical subgroup for malignancy and reduced the calculated NPV. Predictive values are prevalence dependent; the same selection process that increased PPV also reduced NPV. The reported value should therefore be described as the NPV within the surgically verified tertiary cancer-center cohort, not as a population-level estimate.

Repeated FNAs may have further affected the estimates because separate specimens obtained from the same nodule at different time points were analyzed as separate cytologic sampling events. Although each procedure generated an independent specimen and report, repeated observations from the same patient or nodule are statistically correlated and may share the same final histopathologic outcome. This design reflects the original real-world dataset but may overrepresent repeatedly sampled lesions. A future nodule-level study should predefine an index specimen, such as the most recent FNA before surgery, for the primary performance analysis and use statistical methods that account for clustering when repeated samples are retained.

### False-negative diagnoses and sampling limitations

False-negative cases accounted for 3.6% of nodules with postoperative histopathologic follow-up (41/1,137), and the missed diagnosis rate of FNA for thyroid nodules reported in most studies was less than 5% [21, 22, 23]. Sampling-related factors accounted for 35 of the 41 false-negative diagnoses.

Very small malignant foci measuring 3 mm or less were the most frequent sampling-related factor. In FND, a minute carcinoma focus may occupy only a small portion of a substantially larger target nodule. Because of this intranodular heterogeneity, even technically adequate ultrasound-guided FNA may obtain only the surrounding benign follicular nodular tissue and fail to capture malignant cells. Sampling limitations in multinodular thyroid disease have also been reported previously [24]. In the present cohort, this mechanism was specifically observed in some cases in which a minute carcinoma focus was embedded within a larger target nodule in FND. Although the correct target nodule was aspirated and matched to the corresponding resection specimen, the cytologic material contained only the surrounding benign follicular nodular component.

Diffuse stromal fibrosis was another source of inadequate tumor representation. Fibrotic lesions may yield scant cellular material despite repeated passes, and the obtained cells may not reflect the most diagnostically relevant area. Cystic degeneration presents a related problem because aspirated fluid may contain macrophages, debris, and degenerative epithelial cells but few or no viable tumor cells. In large, heterogeneous, or partially cystic lesions, the solid mural component and any sonographically suspicious focal area should therefore be targeted preferentially.

FND may create more than one sampling challenge. A large nodular lesion may contain areas with different architecture and cytologic composition, and a small malignant component may be difficult to identify sonographically within the broader benign nodular background. Multiple passes from spatially distinct regions may improve representation when a target is large or heterogeneous. Nevertheless, no aspiration strategy can guarantee capture of a minute carcinoma focus, and this inherent limitation should be considered when benign cytology is discordant with highly suspicious imaging or clinical findings.

Six false-negative cases were attributed primarily to diagnostic or interpretive factors. Three involved low-risk or follicular-patterned neoplasms whose definitive classification required evaluation of capsular or vascular invasion. This represents a structural limitation of cytology rather than simply an error of observation. Two cases reflected diagnostic oversight, and one involved failure to recognize a malignant component in bilateral nodule aspirates. These findings emphasize the importance of reviewing the complete specimen, correlating each slide with its anatomical target, and obtaining secondary review when cytologic findings are focal, heterogeneous, or discordant with imaging.

#### False-positive diagnoses and cytomorphologic overlap

Most false-positive diagnoses were related to interpretive overlap between benign or reactive follicular epithelial changes and papillary thyroid carcinoma. Follicular epithelial hyperplasia may produce cellular specimens with crowded or overlapping groups, nuclear enlargement, focal chromatin clearing, and irregular nuclear contours. Papillary hyperplasia may form papillary-like groups that raise concern for papillary thyroid carcinoma, particularly when the preparation contains focal nuclear atypia. These findings should be interpreted in the context of the overall architecture, the extent and distribution of nuclear abnormalities, and the accompanying background rather than on the basis of a single feature.

Thyroiditis was another important diagnostic pitfall. Reactive follicular epithelial cells in chronic lymphocytic, Hashimoto, or subacute thyroiditis may show nuclear enlargement, pale chromatin, irregular contours, grooves, and occasionally pseudoinclusion-like changes in an inflammatory background. The combination of reactive atypia and limited cell groups can lead to overclassification when the inflammatory context is not fully appreciated. Similarly, degenerative epithelial cells in cystic lesions may show atypical nuclear changes, and prior radiofrequency ablation can produce treatment-related nuclear enlargement and irregularity. A detailed clinical history is therefore important, particularly when the patient has undergone local treatment.

The solid cell nest case illustrates another uncommon source of error. Cohesive groups of relatively uniform cells with focal nuclear abnormalities may be interpreted as suspicious when the characteristic histologic context is unavailable. Because uncommon benign mimics may not be confidently recognized in cytology alone, category V should be used carefully when the observed features are concerning but incomplete, and expert review may be appropriate before a definitive category VI diagnosis is issued.

One cytology-positive/histology-negative case involved a radiologically identified 3-mm lesion with cytologic features interpreted as papillary thyroid carcinoma, but no corresponding malignant focus was found in the surgical specimen. The available evidence did not establish complete removal by FNA. Failure to identify the lesion during gross examination, incomplete histologic representation, or a localization limitation remained possible. The case is therefore best regarded as an unresolved cytology–histology discrepancy rather than proof of cytologic overdiagnosis or evidence that histopathology should not serve as the reference standard.

### Strategies for reducing diagnostic discordance

Reduction of false-negative and false-positive diagnoses requires coordinated improvement at the stages of lesion selection, specimen acquisition, slide preparation, interpretation, and cytology–histology correlation. Before aspiration, each target nodule should be documented by laterality, anatomical location, dimensions, and ultrasonographic characteristics and assigned a unique identifier. This is especially important in patients with multiple nodules and in FND. For large or heterogeneous nodules, aspiration should preferentially target any focal solid area, mural nodule, or region with suspicious ultrasonographic features. Multiple spatially separate passes may reduce sampling bias, while recognizing that a minute carcinoma focus may still remain unsampled.

For cystic or partially cystic lesions, aspiration of cyst fluid alone is often insufficient; the residual solid component or mural nodule should be sampled. Rapid on-site evaluation, when available, can identify hypocellular or nondiagnostic material and permit immediate additional passes. Where rapid on-site evaluation is not available, adequate pass numbers, careful liquid-based cytology preparation, and appropriate use of residual material for cell-block preparation may improve diagnostic yield. Repeat ultrasound-guided FNA should be considered after a category I result and when benign cytology is discordant with highly suspicious imaging, interval growth, or other clinical findings.

Interpretive strategies are equally important. Isolated nuclear enlargement, chromatin clearing, grooves, or occasional pseudoinclusions should not be interpreted as malignant without considering their extent, distribution, architecture, and background. Inflammatory, cystic, hyperplastic, and treatment-related conditions should be actively considered. When atypia is limited or equivocal, classification as AUS may be more appropriate than suspicious for malignancy or malignant. The 2023 TBSRTC subdivision of AUS into nuclear atypia and other atypia may improve risk stratification, although this could not be evaluated retrospectively in the present cohort.

Difficult cases may benefit from secondary review by an experienced cytopathologist with access to the relevant ultrasound images and clinical history. Selective molecular testing may assist management of indeterminate nodules when the result is likely to change treatment, although such testing was not routinely available in this cohort. Regular multidisciplinary review of discordant cases can identify recurrent sampling and interpretive pitfalls and can improve communication among ultrasonographers, cytopathologists, surgeons, and histopathologists. Strict one-to-one matching of the aspirated target with the corresponding histologic lesion is essential for meaningful ROM and diagnostic-performance calculations.

#### Borderline and low-risk thyroid neoplasms

The 2022 WHO classification recognizes a family of low-risk follicular cell-derived neoplasms that includes NIFTP, tumors of uncertain malignant potential, and hyalinizing trabecular tumor [7]. The present cohort contained 13 NIFTPs, 4 FT-UMPs, and 5 WDT-UMPs; no HTT was identified. These entities were grouped operationally for ROM analysis, but they are not biologically identical and should not be described collectively as carcinoma.

Category V was the most frequent cytologic category among the 22 low-risk neoplasms, followed by categories III and II. Some lesions subsequently classified as NIFTP showed a predominantly follicular architecture accompanied by sufficiently developed papillary thyroid carcinoma-like nuclear features to support a suspicious-for-malignancy interpretation. However, they lacked sufficiently unequivocal features for category VI. This distribution reflects institutional interpretive thresholds and does not imply that NIFTP can be diagnosed specifically by FNA.

A definitive diagnosis of NIFTP requires complete histologic examination of the tumor-capsule interface to exclude capsular or vascular invasion and to confirm that the lesion meets the full architectural criteria. Similarly, cytology cannot distinguish FT-UMP, WDT-UMP, and follicular carcinoma when the decisive feature is invasion. The earlier statement implying that capsular invasion is not compatible with carcinoma was therefore incorrect. Rosai’s discussion distinguished minimally invasive from widely invasive follicular carcinoma and emphasized differences in prognosis; it did not remove unequivocally invasive lesions from the carcinoma category [25]. The 2022 WHO classification continues to recognize invasive encapsulated follicular-patterned carcinomas. Borderline or low-risk neoplasms accounted for 22 of the 1,137 nodules with histopathologic follow-up. Their inclusion as positive outcomes increased ROM1 compared with ROM2, but the magnitude of the change varied by category. The largest absolute differences occurred in categories containing a greater proportion of these lesions, particularly categories III and V. This finding supports reporting both analytical definitions when comparing historical data, because the classification of NIFTP and tumors of uncertain malignant potential can materially alter category-specific ROM [26]. At the same time, the analytical grouping should not be interpreted as indicating that these lesions are biologically equivalent to carcinoma. Indeterminate follicular-patterned lesions may benefit from multidisciplinary review integrating cytologic, ultrasonographic, and clinical findings to estimate the risk of malignancy and guide patient management. Molecular testing of FNA specimens classified as Bethesda category III (AUS) or IV (FN) may provide additional information for risk stratification and surgical decision-making; however, it cannot establish a definitive diagnosis of NIFTP [27]. A diagnosis of NIFTP requires histopathologic examination of the resected lesion, including complete evaluation of the tumor–capsule interface to exclude capsular or vascular invasion. Although frozen-section examination may occasionally demonstrate unequivocal invasion, its diagnostic sensitivity is limited because capsular or vascular invasion may be focal. Therefore, a negative or deferred frozen-section result cannot reliably exclude carcinoma, and neither cytology nor frozen-section examination can establish a definitive diagnosis of NIFTP or a tumor of uncertain malignant potential [28, 29].

#### Strengths and limitations

The principal strengths of this study are the large number of thyroid FNA specimens, the availability of postoperative histopathology for more than one thousand aspirated nodules, the category-specific analysis of low-risk neoplasms, and the detailed retrospective review of false-negative and false-positive cases. The study also demonstrates the practical value of separating sampling limitations from interpretive factors and of distinguishing strict target-nodule matching from unrelated incidental malignancy.

Several limitations remain. First, the study was retrospective and conducted at a single tertiary cancer hospital. Referral bias produced a high prevalence of malignant disease, and the results may not be generalizable to general hospitals or screening populations. Second, histopathologic confirmation was available for 49.1% of FNA specimens. Surgery was determined by routine clinical care rather than a standardized protocol, and the verified cohort was affected by surgical selection and partial verification. These biases likely inflated the observed ROM in categories I–III and reduced the NPV.

Third, repeated FNAs from the same nodule at different time points were treated as separate sampling events. These observations are correlated and may share the same histopathologic outcome. Fourth, standardized nodule-size and ultrasonographic variables, including composition, echogenicity, margins, calcifications, shape, and TI-RADS category, were not consistently available for the entire diagnostic-performance cohort. The study could therefore describe the 28 false-negative cases with malignant foci measuring 3 mm or less but could not provide valid size-stratified sensitivity or accuracy because corresponding denominators for all true-positive, true-negative, false-positive, and false-negative cases were unavailable.

Fifth, systematic retrospective slide review was limited to discordant cases. Concordant cases were not comprehensively re-evaluated, and residual diagnostic misclassification cannot be excluded. The original diagnoses predated the 2023 TBSRTC, so the recommended AUS subclassification into nuclear atypia and other atypia could not be applied reliably. The 2022 WHO classification was also not implemented consistently throughout the entire study period; consequently, invasive encapsulated follicular variant papillary thyroid carcinoma was not recorded as a separate category and may have been included in the broader FVPTC group.

Finally, molecular testing and long-term clinical or imaging follow-up were not routinely available. Formal interobserver agreement could not be assessed because the two cytopathologists interpreted mutually exclusive case sets rather than independently reviewing the same specimens. Future multicenter prospective studies should use standardized ultrasound reporting, unique target-nodule identifiers, predefined handling of repeated FNA, comprehensive slide review, and long-term follow-up.

## Conclusion

In this surgically selected tertiary cancer-center cohort, thyroid FNAC classified using the Bethesda framework showed high sensitivity, specificity, accuracy, and PPV. Sampling limitations involving minute malignant foci, FND, fibrosis, cystic degeneration, and lesion heterogeneity were the principal causes of false-negative diagnoses, whereas reactive, inflammatory, hyperplastic, cystic, and treatment-related changes were the main sources of false-positive interpretation. Accurate ultrasound-guided targeting, representative sampling, cautious interpretation of focal atypia, and strict nodule-level cytology–histology correlation may improve diagnostic performance. The reported ROM and predictive values should be interpreted cautiously because of referral bias, surgical selection, partial verification, and the retrospective study design.

## Data Availability

The datasets used and/or analysed during the current study available from the corresponding author on reasonable request.

## Abbreviations

FNAC: Fine-needle aspiration cytology
FNA: Fine-needle aspiration
TBSRTC: The Bethesda System for Reporting Thyroid Cytopathology
ROM: Risk of malignancy
WHO: World Health Organization
AUS: Atypia of undetermined significance
NIFTP: Non-invasive follicular thyroid neoplasm with papillary-like nuclear features
FT-UMP: Follicular tumor of uncertain malignant potential
WDT-UMP: Well-differentiated tumor of uncertain malignant potential
PPV: Positive predictive value
NPV: Negative predictive value
CVPTC: Classic papillary thyroid carcinoma
FVPTC: Follicular variant papillary thyroid carcinoma
PTC: Papillary thyroid carcinoma
FTC: Follicular thyroid carcinoma
MTC: Medullary thyroid carcinoma
ATC: Anaplastic thyroid carcinoma
FND: Follicular nodular disease
